# 2,2,7,7-Tetra­methyl-1,2,3,6,7,8-hexa­hydro­cinnolino[5,4,3-*cde*]cinnoline

**DOI:** 10.1107/S1600536808043912

**Published:** 2009-01-08

**Authors:** Ju-Hua Peng, Wen-Juan Hao, Shu-Jiang Tu

**Affiliations:** a Lianyungang Teachers’ College, Lianyungang 222006, People’s Republic of China; b School of Chemistry and Chemical Engineering, Xuzhou Normal University, Xuzhou 221116, People’s Republic of China

## Abstract

The asymmetric unit of the title compound, C_16_H_20_N_4_, contains two half-mol­ecules, which are completed by crystallographic inversion symmetry. The pyridazine rings are conjugated and the cyclo­hexane rings adopt envelope conformations.

## Related literature

For general background, see: Ischikawa *et al.* (1992 ▶); Labovitz *et al.* (1990 ▶); Mizutani, Shiroshita, Okuda *et al.* (1989 ▶); Patterson (1992 ▶); Coghlan *et al.* (1989 ▶); Mizutani, Shiroshita, Sakaki *et al.* (1989 ▶b); Munro & Bit (1987 ▶); Inoue *et al.* (1993 ▶); Tutsumi *et al.* (1992 ▶); Yokomoto *et al.* (1992 ▶); Miyamoto *et al.* (1990 ▶). For bond-length data, see: Allen *et al.* (1987 ▶). For ring-puckering parameters, see: Cremer & Pople (1975 ▶).
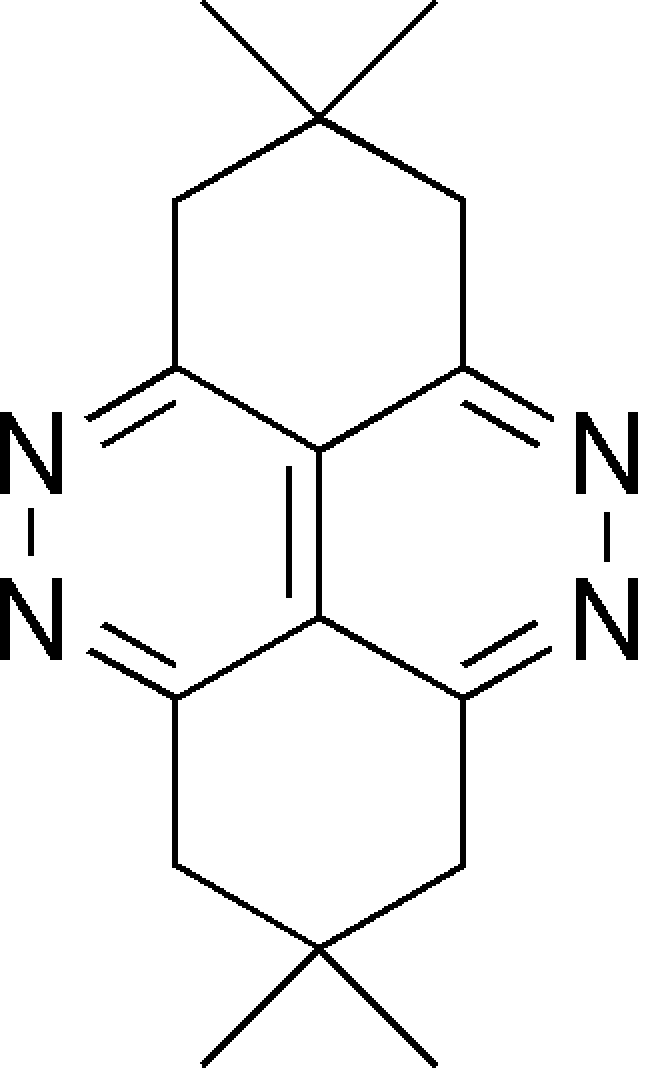


## Experimental

### 

#### Crystal data

C_16_H_20_N_4_
*M* *_r_* = 268.36
Monoclinic, 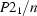

*a* = 12.819 (4) Å
*b* = 8.441 (3) Å
*c* = 13.310 (4) Å
β = 95.462 (5)°
*V* = 1433.7 (8) Å^3^
*Z* = 4
Mo *K*α radiation
μ = 0.08 mm^−1^
*T* = 298 (2) K
0.33 × 0.28 × 0.21 mm

#### Data collection

Bruker SMART CCD area-detector diffractometer
Absorption correction: multi-scan (*SADABS*; Bruker, 1998 ▶) *T*_min_ = 0.975, *T*_max_ = 0.984
7218 measured reflections
2518 independent reflections
1416 reflections with *I* > 2σ(*I*)
*R*_int_ = 0.048

#### Refinement

*R*[*F* ^2^ > 2σ(*F* ^2^)] = 0.052
*wR*(*F* ^2^) = 0.179
*S* = 1.04
2518 reflections
185 parameters
H-atom parameters constrained
Δρ_max_ = 0.18 e Å^−3^
Δρ_min_ = −0.20 e Å^−3^

### 

Data collection: *SMART* (Bruker, 1998 ▶); cell refinement: *SAINT* (Bruker, 1998 ▶); data reduction: *SAINT*; program(s) used to solve structure: *SHELXS97* (Sheldrick, 2008 ▶); program(s) used to refine structure: *SHELXL97* (Sheldrick, 2008 ▶); molecular graphics: *SHELXTL* (Sheldrick, 2008 ▶); software used to prepare material for publication: *SHELXTL*.

## Supplementary Material

Crystal structure: contains datablocks global, I. DOI: 10.1107/S1600536808043912/hk2606sup1.cif

Structure factors: contains datablocks I. DOI: 10.1107/S1600536808043912/hk2606Isup2.hkl

Additional supplementary materials:  crystallographic information; 3D view; checkCIF report

## Figures and Tables

**Fig. 1.**
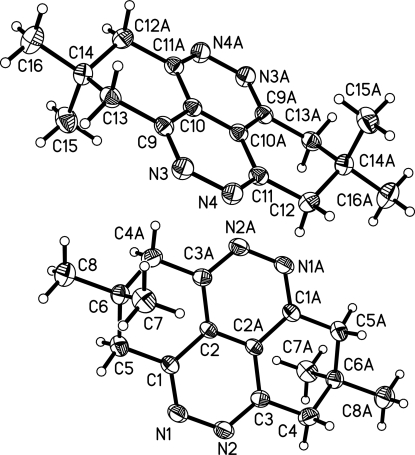
The molecular structure of the title molecule, with the atom-numbering scheme. Displacement ellipsoids are drawn at the 50% probability level [symmetry code (A): -x, -y, -z].

## supplementary crystallographic information

### Comment

It is well known that six-membered nitrogen-containing heterocycles are abundant in numerous natural products that exhibit important biological properties. For example, cinnolines and their derivatives are widely used as agrochemical and pharmaceutical drugs (Ischikawa *et al.*,  1992; Labovitz *et al.*,  1990; Mizutani, Shiroshita, Okuda *et al.*, 1989; Patterson, 1992; Coghlan *et al.*, 1989; Mizutani, Shiroshita, Sakaki *et al.*, 1989; Munro & Bit, 1987). They can act as microbicides, pollen suppressants, fungicides and herbicides in agrochemistry. They can also be used as bactericides in pharmaceutical industry (Inoue *et al.*,  1993; Tutsumi *et al.*,  1992; Yokomoto *et al.*,  1992; Miyamoto *et al.*, 1990). The chemistry of cinnolines has received much attention based on the above facts.

The asymmetric unit of the title compound contains two-halves of centrosymmetric molecules (Fig. 1). The bond lengths (Allen *et al.*,  1987) and angles are within normal ranges. The pyridazine rings A (N1/N2/C1-C3/C2A) and C (N3/N4/C9-C11/C10B) are, of course, planar and they are oriented at a dihedral angle of 43.89 (3)° [symmetry codes: (A) 2 - x, -y, -z; (B) 1 - x, -y, -z]. The cyclohexene rings B (C1/C2/C5/C6/C3A/C4A) and D (C9/C10/C13/C14/C11B/C12B), having total puckering amplitudes, Q_T_, of 0.579 (3) and 0.566 Å, respectively, half-chair conformations [φ = -72.92 (3)° and θ = 103.84 (4)°; φ = 110.31 (4)° and θ = 74.14 (4)°] (Cremer & Pople, 1975) [symmetry codes: (A) 2 - x, -y, -z; (B) 1 - x, -y, -z].

### Experimental

The title compound was prepared by the reaction of 3,4,6,7-tetrahydro- 3,3,6,6,9-pentamethyl-2*H*-xanthene-1,8(5*H*,9H)-dione (2 mmol) and hydrazine hydrate (8 mmol, 80%) in ethanol (8 ml), stirring at 353 K (yield; 88%, m.p. 562-563 K). Crystals suitable for X-ray analysis were obtained from an ethanol solution by slow evaporation.

### Refinement

H atoms were positioned geometrically, with C-H = 0.97 and 0.96 Å for methylene and methyl H and constrained to ride on their parent atoms, with U_iso_(H) = xU_eq_(C), where x = 1.5 for methyl H and x = 1.2 for methylene H atoms.

### Crystal data

**Table:**

C_16_H_20_N_4_	*F*(000) = 576
*M**_r_* = 268.36	*D*_x_ = 1.243 Mg m^−^^3^
Monoclinic, *P*2_1_/*n*	Melting point = 562–563 K
Hall symbol: -P 2yn	Mo *K*α radiation, λ = 0.71073 Å
*a* = 12.819 (4) Å	Cell parameters from 1230 reflections
*b* = 8.441 (3) Å	θ = 2.3–23.2°
*c* = 13.310 (4) Å	µ = 0.08 mm^−^^1^
β = 95.462 (5)°	*T* = 298 K
*V* = 1433.7 (8) Å^3^	Block, pale yellow
*Z* = 4	0.33 × 0.28 × 0.21 mm

### Data collection

**Table:**

Bruker SMART CCD area-detector diffractometer	2518 independent reflections
Radiation source: fine-focus sealed tube	1416 reflections with *I* > 2σ(*I*)
graphite	*R*_int_ = 0.048
φ and ω scans	θ_max_ = 25.0°, θ_min_ = 2.1°
Absorption correction: multi-scan (*SADABS*; Bruker, 1998)	*h* = −15→15
*T*_min_ = 0.975, *T*_max_ = 0.984	*k* = −9→10
7218 measured reflections	*l* = −15→14

### Refinement

**Table:**

Refinement on *F*^2^	Primary atom site location: structure-invariant direct methods
Least-squares matrix: full	Secondary atom site location: difference Fourier map
*R*[*F*^2^ > 2σ(*F*^2^)] = 0.052	Hydrogen site location: inferred from neighbouring sites
*wR*(*F*^2^) = 0.179	H-atom parameters constrained
*S* = 1.04	*w* = 1/[σ^2^(*F*_o_^2^) + 0.3048*P*] where *P* = (*F*_o_^2^ + 2*F*_c_^2^)/3
2518 reflections	(Δ/σ)_max_ < 0.001
185 parameters	Δρ_max_ = 0.18 e Å^−^^3^
0 restraints	Δρ_min_ = −0.20 e Å^−^^3^

### Special details

**Table:**

Geometry. All e.s.d.'s (except the e.s.d. in the dihedral angle between two l.s. planes) are estimated using the full covariance matrix. The cell e.s.d.'s are taken into account individually in the estimation of e.s.d.'s in distances, angles and torsion angles; correlations between e.s.d.'s in cell parameters are only used when they are defined by crystal symmetry. An approximate (isotropic) treatment of cell e.s.d.'s is used for estimating e.s.d.'s involving l.s. planes.
Refinement. Refinement of *F*^2^ against ALL reflections. The weighted *R*-factor *wR* and goodness of fit *S* are based on *F*^2^, conventional *R*-factors *R* are based on *F*, with *F* set to zero for negative *F*^2^. The threshold expression of *F*^2^ > σ(*F*^2^) is used only for calculating *R*-factors(gt) *etc*. and is not relevant to the choice of reflections for refinement. *R*-factors based on *F*^2^ are statistically about twice as large as those based on *F*, and *R*- factors based on ALL data will be even larger.

### Fractional atomic coordinates and isotropic or equivalent isotropic displacement parameters (Å^2^)

**Table:**

	*x*	*y*	*z*	*U*_iso_*/*U*_eq_	
N1	1.11488 (18)	0.2369 (3)	0.00759 (19)	0.0510 (7)	
N2	1.16426 (18)	0.1239 (3)	−0.04672 (19)	0.0513 (7)	
N3	0.50653 (19)	0.2577 (3)	−0.08920 (17)	0.0469 (6)	
N4	0.56253 (19)	0.1517 (3)	−0.14224 (17)	0.0470 (6)	
C1	1.0250 (2)	0.2044 (3)	0.04438 (19)	0.0394 (7)	
C2	0.97626 (18)	0.0553 (3)	0.02796 (18)	0.0350 (7)	
C3	1.1205 (2)	−0.0146 (3)	−0.06568 (19)	0.0408 (7)	
C4	1.1709 (2)	−0.1336 (3)	−0.1275 (2)	0.0522 (8)	
H4A	1.2463	−0.1185	−0.1187	0.063*	
H4B	1.1479	−0.1161	−0.1982	0.063*	
C5	0.9742 (2)	0.3236 (4)	0.1053 (2)	0.0483 (8)	
H5A	0.9906	0.4287	0.0819	0.058*	
H5B	1.0030	0.3146	0.1752	0.058*	
C6	0.8543 (2)	0.3047 (3)	0.0998 (2)	0.0456 (7)	
C7	0.8054 (2)	0.3422 (4)	−0.0065 (2)	0.0602 (9)	
H7A	0.8324	0.2704	−0.0536	0.090*	
H7B	0.8224	0.4490	−0.0237	0.090*	
H7C	0.7307	0.3308	−0.0091	0.090*	
C8	0.8112 (3)	0.4183 (4)	0.1746 (3)	0.0705 (10)	
H8A	0.7364	0.4078	0.1711	0.106*	
H8B	0.8290	0.5251	0.1582	0.106*	
H8C	0.8411	0.3935	0.2416	0.106*	
C9	0.4617 (2)	0.2110 (3)	−0.00971 (19)	0.0380 (7)	
C10	0.47112 (19)	0.0524 (3)	0.02548 (18)	0.0361 (7)	
C11	0.5739 (2)	0.0028 (3)	−0.11235 (19)	0.0386 (7)	
C12	0.6346 (2)	−0.1106 (3)	−0.1700 (2)	0.0460 (8)	
H12A	0.7083	−0.1023	−0.1463	0.055*	
H12B	0.6267	−0.0816	−0.2408	0.055*	
C13	0.3987 (2)	0.3244 (3)	0.0462 (2)	0.0443 (7)	
H13A	0.3267	0.3235	0.0162	0.053*	
H13B	0.4261	0.4306	0.0394	0.053*	
C14	0.4010 (2)	0.2827 (3)	0.1591 (2)	0.0448 (8)	
C15	0.5115 (3)	0.3042 (4)	0.2106 (2)	0.0646 (10)	
H15A	0.5326	0.4127	0.2047	0.097*	
H15B	0.5590	0.2368	0.1790	0.097*	
H15C	0.5124	0.2767	0.2807	0.097*	
C16	0.3261 (3)	0.3920 (4)	0.2079 (2)	0.0652 (10)	
H16A	0.3267	0.3663	0.2782	0.098*	
H16B	0.2566	0.3788	0.1754	0.098*	
H16C	0.3480	0.5000	0.2010	0.098*	

### Atomic displacement parameters (Å^2^)

**Table:**

	*U*^11^	*U*^22^	*U*^33^	*U*^12^	*U*^13^	*U*^23^
N1	0.0418 (15)	0.0440 (15)	0.0675 (17)	−0.0085 (12)	0.0070 (13)	−0.0044 (13)
N2	0.0442 (14)	0.0409 (15)	0.0702 (17)	−0.0060 (12)	0.0122 (12)	−0.0011 (13)
N3	0.0583 (16)	0.0392 (14)	0.0445 (14)	−0.0001 (12)	0.0117 (12)	0.0062 (11)
N4	0.0593 (16)	0.0392 (15)	0.0442 (14)	−0.0027 (12)	0.0133 (12)	0.0036 (11)
C1	0.0385 (16)	0.0370 (17)	0.0416 (16)	−0.0029 (13)	−0.0025 (13)	0.0016 (13)
C2	0.0322 (15)	0.0314 (15)	0.0402 (16)	−0.0019 (11)	−0.0026 (12)	0.0033 (11)
C3	0.0413 (16)	0.0387 (17)	0.0424 (16)	−0.0017 (14)	0.0045 (13)	0.0057 (13)
C4	0.0490 (18)	0.050 (2)	0.0595 (19)	0.0021 (15)	0.0164 (15)	0.0029 (15)
C5	0.0491 (18)	0.0458 (18)	0.0488 (17)	−0.0039 (15)	−0.0014 (14)	−0.0070 (14)
C6	0.0457 (17)	0.0406 (17)	0.0507 (17)	0.0024 (14)	0.0067 (14)	−0.0028 (14)
C7	0.055 (2)	0.052 (2)	0.071 (2)	0.0062 (16)	−0.0062 (16)	0.0042 (17)
C8	0.068 (2)	0.061 (2)	0.084 (3)	0.0038 (19)	0.0189 (19)	−0.0165 (19)
C9	0.0411 (16)	0.0354 (17)	0.0370 (15)	−0.0034 (13)	0.0004 (12)	0.0020 (13)
C10	0.0367 (15)	0.0380 (17)	0.0331 (15)	−0.0046 (12)	0.0008 (11)	0.0016 (11)
C11	0.0412 (16)	0.0380 (17)	0.0366 (15)	−0.0045 (13)	0.0033 (12)	0.0039 (13)
C12	0.0470 (17)	0.0492 (19)	0.0429 (16)	0.0018 (14)	0.0091 (13)	0.0031 (14)
C13	0.0459 (17)	0.0349 (16)	0.0518 (17)	−0.0025 (13)	0.0030 (13)	0.0026 (14)
C14	0.0489 (18)	0.0416 (18)	0.0438 (16)	0.0031 (14)	0.0053 (14)	−0.0027 (13)
C15	0.064 (2)	0.061 (2)	0.065 (2)	−0.0014 (18)	−0.0099 (17)	−0.0108 (18)
C16	0.081 (2)	0.060 (2)	0.057 (2)	0.0161 (19)	0.0187 (18)	−0.0003 (17)

### Geometric parameters (Å, °)

**Table:**

N1—C1	1.322 (3)	C8—H8A	0.9600
N1—N2	1.386 (3)	C8—H8B	0.9600
N2—C3	1.311 (3)	C8—H8C	0.9600
N3—C9	1.312 (3)	C9—C10	1.420 (4)
N3—N4	1.382 (3)	C9—C13	1.495 (4)
N4—C11	1.323 (4)	C10—C10^ii^	1.373 (5)
C1—C2	1.413 (3)	C10—C11^ii^	1.419 (3)
C1—C5	1.482 (4)	C11—C10^ii^	1.419 (3)
C2—C2^i^	1.371 (5)	C11—C12	1.491 (4)
C2—C3^i^	1.423 (3)	C12—C14^ii^	1.534 (4)
C3—C2^i^	1.423 (3)	C12—H12A	0.9700
C3—C4	1.485 (4)	C12—H12B	0.9700
C4—C6^i^	1.533 (4)	C13—C14	1.541 (4)
C4—H4A	0.9700	C13—H13A	0.9700
C4—H4B	0.9700	C13—H13B	0.9700
C5—C6	1.540 (4)	C14—C16	1.521 (4)
C5—H5A	0.9700	C14—C15	1.525 (4)
C5—H5B	0.9700	C14—C12^ii^	1.534 (4)
C6—C8	1.524 (4)	C15—H15A	0.9600
C6—C7	1.525 (4)	C15—H15B	0.9600
C6—C4^i^	1.533 (4)	C15—H15C	0.9600
C7—H7A	0.9600	C16—H16A	0.9600
C7—H7B	0.9600	C16—H16B	0.9600
C7—H7C	0.9600	C16—H16C	0.9600
			
C1—N1—N2	120.5 (2)	H8A—C8—H8C	109.5
C3—N2—N1	120.4 (2)	H8B—C8—H8C	109.5
C9—N3—N4	120.4 (2)	N3—C9—C10	121.3 (2)
C11—N4—N3	120.6 (2)	N3—C9—C13	120.5 (2)
N1—C1—C2	121.0 (2)	C10—C9—C13	118.2 (2)
N1—C1—C5	120.4 (2)	C10^ii^—C10—C11^ii^	118.1 (3)
C2—C1—C5	118.6 (2)	C10^ii^—C10—C9	118.5 (3)
C2^i^—C2—C1	118.5 (3)	C11^ii^—C10—C9	123.4 (2)
C2^i^—C2—C3^i^	118.3 (3)	N4—C11—C10^ii^	121.0 (2)
C1—C2—C3^i^	123.2 (2)	N4—C11—C12	120.2 (2)
N2—C3—C2^i^	121.2 (2)	C10^ii^—C11—C12	118.8 (2)
N2—C3—C4	120.5 (2)	C11—C12—C14^ii^	112.6 (2)
C2^i^—C3—C4	118.2 (2)	C11—C12—H12A	109.1
C3—C4—C6^i^	113.0 (2)	C14^ii^—C12—H12A	109.1
C3—C4—H4A	109.0	C11—C12—H12B	109.1
C6^i^—C4—H4A	109.0	C14^ii^—C12—H12B	109.1
C3—C4—H4B	109.0	H12A—C12—H12B	107.8
C6^i^—C4—H4B	109.0	C9—C13—C14	112.2 (2)
H4A—C4—H4B	107.8	C9—C13—H13A	109.2
C1—C5—C6	113.1 (2)	C14—C13—H13A	109.2
C1—C5—H5A	109.0	C9—C13—H13B	109.2
C6—C5—H5A	109.0	C14—C13—H13B	109.2
C1—C5—H5B	109.0	H13A—C13—H13B	107.9
C6—C5—H5B	109.0	C16—C14—C15	109.4 (3)
H5A—C5—H5B	107.8	C16—C14—C12^ii^	109.2 (2)
C8—C6—C7	109.5 (3)	C15—C14—C12^ii^	110.0 (2)
C8—C6—C4^i^	109.7 (2)	C16—C14—C13	108.9 (2)
C7—C6—C4^i^	110.0 (2)	C15—C14—C13	110.0 (2)
C8—C6—C5	109.0 (2)	C12^ii^—C14—C13	109.3 (2)
C7—C6—C5	110.1 (2)	C14—C15—H15A	109.5
C4^i^—C6—C5	108.6 (2)	C14—C15—H15B	109.5
C6—C7—H7A	109.5	H15A—C15—H15B	109.5
C6—C7—H7B	109.5	C14—C15—H15C	109.5
H7A—C7—H7B	109.5	H15A—C15—H15C	109.5
C6—C7—H7C	109.5	H15B—C15—H15C	109.5
H7A—C7—H7C	109.5	C14—C16—H16A	109.5
H7B—C7—H7C	109.5	C14—C16—H16B	109.5
C6—C8—H8A	109.5	H16A—C16—H16B	109.5
C6—C8—H8B	109.5	C14—C16—H16C	109.5
H8A—C8—H8B	109.5	H16A—C16—H16C	109.5
C6—C8—H8C	109.5	H16B—C16—H16C	109.5

Symmetry codes: (i) −*x*+2, −*y*, −*z*; (ii) −*x*+1, −*y*, −*z*.
